# Magnitude and Determinants of Perinatal Mortality in Southwest Ethiopia

**DOI:** 10.1155/2020/6859157

**Published:** 2020-09-22

**Authors:** Gurmesa Tura Debelew

**Affiliations:** Department of Population and Family Health, Faculty of Public Health, Institute of Health, Jimma University, Jimma, P.O. Box: 378, Ethiopia

## Abstract

Despite several efforts globally, the problem of perinatal mortality remained an unsolved agenda. As a result, it continued to be an essential part of the third sustainable development goals to end preventable child deaths by 2030. With a rate of 33 per 1000 births, Ethiopia has the highest level of perinatal mortality in the world. Thus, determining the magnitude and identifying the determinants are very crucial for evidence-based interventions. A community-based longitudinal study was conducted in Southwest Ethiopia among 3474 pregnant women to estimate the magnitude of perinatal mortality. Then, a case-control study among 120 cases and 360 controls was conducted to identify the determinants of perinatal mortality. Data were collected by using an interviewer-administered questionnaire and analyzed by using SPSS version 20. Multivariate logistic regression analysis was used to identify variables having a significant association with perinatal mortality at *p* < 0.05. The perinatal mortality rate was 34.5 (95% CI: 28.9, 41.1) deaths per 1000 births. Attending ≥4 ANC visits (AOR = 0.46; 95% CI: 0.23, 0.91), having good knowledge on key danger signs (AOR = 0.27; 95% CI: 0.10, 0.75), and having a skilled attendant at birth (AOR = 0.34; 95% CI: 0.19, 0.61) were significantly associated with a reduction of perinatal mortality. Being a primipara (AOR = 3.38; 95% CI: 1.90, 6.00), twin births (AOR = 5.29; 95% CI: 1.46, 19.21), previous history of perinatal mortality (AOR = 3.33; 95% CI: 1.27, 8.72), and obstetric complication during labor (AOR = 4.27; 95% CI: 2.40, 7.59) significantly increased perinatal mortality. In conclusion, the magnitude of perinatal mortality in the study area was high as compared to the national target for 2020. Care during pregnancy and childbirth and conditions of pregnancy and labor were identified as determinants of perinatal mortality. Hence, interventions need to focus on increasing knowledge of danger signs and utilization of skilled maternity care. Special emphasis needs to be given to mothers with a previous history of perinatal mortality, twin pregnancies, and having obstetric complications.

## 1. Introduction

According to the World Health Organization (WHO), perinatal mortality refers to the death of a fetus after 28 completed weeks of gestation (stillbirth) plus early neonatal death within the first seven days of birth [1]. This perinatal period is the most critical period in an individual's life, and the rate of death during this period is higher than any other period of life. Perinatal mortality (both stillbirth and early neonatal death) is particularly related to the maternal conditions during late pregnancy and intrapartum conditions and reflects the quality of delivery care. Perinatal mortality is also a key indicator of socioeconomic development and the overall health status of any country [1].

With more than 5 million perinatal deaths occurring worldwide every year, ending preventable perinatal deaths will continue to be a significant part of the global public health agenda beyond 2015. As a result, reducing stillbirths and early neonatal deaths continued to be an essential part of the third Sustainable Development Goal (SDG-3), to end preventable child deaths by 2030 [2, 3].

Perinatal mortality is a major public health problem, particularly in developing countries, and has a huge economic, social, and health implications for families and nations. This is because more than 95% of perinatal deaths occur in developing countries with the largest numbers being in South Asia and Sub-Saharan Africa (SSA) [4].

With a perinatal mortality rate of 34.7 per 1000 births, Sub-Saharan Africa (SSA) has one of the highest levels of perinatal mortality in the world. Unless special emphasis is given to improving access to high-quality health services during pregnancy and childbirth as well as strengthening health care systems, the attainment of the global sustainable development goal related to neonatal and child mortality may be challenging for the countries in Sub-Saharan Africa [4].

Ethiopia is one of the fast track sub-Saharan African countries in reducing under-five mortality. However, the reduction in neonatal and perinatal mortality continues to be a major challenge, and with a rate of 33 deaths per 1000 births, Ethiopia is one of the countries with the highest perinatal mortality rate in the world [5]. Though community-based studies are limited in Southwest Ethiopia, a study conducted in Jimma University Specialized Hospital has shown that the facility-based perinatal mortality rate was as high as 98.2 per 1000 births, which is higher than any figure in the country [6].

To achieve the national target of reducing both stillbirth and neonatal mortality to less than 10 per 1000 by 2020 [7] and contribute to the attainment of the global sustainable development goal, a clear knowledge of the determinants of perinatal mortality at a local context is very crucial. Besides, identifying the determinants is very essential for the prioritization of local initiatives, which focus on encouraging evidence-based advocacy and effective interventions targeting perinatal mortality reduction, through local decision-making. But most of the previous studies have been limited to facility-based reporting of the burden of stillbirth and neonatal mortality [6], and community-based studies identifying the determinants at a large scale are limited.

Hence, this study is aimed at filling this gap by conducting a community-based study to determine the status of perinatal mortality and identify the determinant factors. It is also hoped that the findings of the study will have paramount importance in guiding the designing of effective interventions and programs to accelerate progress in perinatal mortality reduction in Southwest Ethiopia in particular and in Ethiopia in general.

## 2. Materials and Methods

### 2.1. Study Area, Period, and Design

This is a community-based study conducted in Jimma Zone, Southwest Ethiopia, as part of a project which started in June 2013 and continued till February 2015. The project was designed to assess the effect of birth preparedness and complication readiness on maternal and perinatal outcomes. The first part of maternal health care was published in *Reproductive Health Journal* [8]. Jimma Zone is one of the 18 administrative zones of the Oromia Regional State of Ethiopia, located 352 km southwest of Addis Ababa, the capital of Ethiopia. The zone has a total of 17 rural districts and two town administrations with a total of 46 urban and 512 rural “kebeles” (the smallest formal administrative units). As projected from the 2007 national population and housing census for the year 2019, the zone has a total population of about 3.2 million, of which 88.7% were rural residents.

### 2.2. Population, Sample Size, and Sampling Methods

The populations for this study were all women, who gave birth during the data collection period in the study area, Jimma Zone. The source population for the cases was all perinatal deaths (stillbirth plus early neonatal death, death in the first week of life) happening during the study period in the study area, and the source population for controls was all neonates who survived the first seven days of life in the same period in the same area.

The sample size to determine the status of the perinatal mortality rate was calculated by using Epi-Info V.3.5.5 considering a single population proportion based on the following assumptions. The prevalence of perinatal mortality was assumed to be 33 per 1000 births (*p* = 3.3%) [5]. In addition, a 95% level of confidence, a 1% margin of error, a 10% nonresponse rate, and a design effect of 2 were considered. This gave a sample size of 2697 births. However, as this study was a part of a big longitudinal study in which 3474 pregnant women were on follow-up, all of them were included in the analysis. The detailed sample size and sampling method of the longitudinal study were published in *Reproductive Health* 2014;11:60 [8].

The sample size for the case-control study was calculated by using Epi-info V.3.5.5 by considering an unmatched case-control study design based on the following assumptions. Adequate antenatal care (ANC4+) visits during pregnancy were taken as an exposure variable for the perinatal mortality. Based on this, the proportion of exposure among controls (percentage of pregnant women not having adequate ANC visits among those who survived the early neonatal period) was assumed to be 63% with an odds ratio (OR) of 3.6 [9]. In addition, a 95% level of confidence, 90% power, case to control ratio of 1 : 3, a design effect of 2, and a 10% nonresponse rate were considered. Based on these assumptions, the final sample size became 512 births (128 cases and 384 controls). Similarly, in the longitudinal study, 120 perinatal deaths were registered and all were taken as cases, and 360 controls were selected from those births surviving the early neonatal period by using the SPSS “select cases” command.

### 2.3. Data Collection Tool, Procedure, and Quality Control

Pretested interviewer-administered structured questionnaire, which was developed after reviewing different related literature, was used to collect the data. The questionnaire was first prepared in English, and then translated to the local language (Afan Oromoo), and then translated back to English to check its consistency. The questionnaire included the basic sociodemographic and economic characteristics, previous reproductive history of the mothers, and possible determinants of perinatal mortality, including maternal characteristics, service use during pregnancy and delivery, and existence of complications during pregnancy and delivery. The data were collected through house-to-house visits by experienced 10th grade completed females who were trained for three days on the objectives and procedures of data collection. The data collection process was strictly supervised by BSc degree holders to ensure its quality.

### 2.4. Operational Definitions and Measurements

*Perinatal mortality*: the death of a fetus after 28 completed weeks of gestation (stillbirth) plus early neonatal death (death within the first seven days of birth).

*Wealth tertiles*: using 18 questions adapted from the Ethiopian Demographic and Health Survey (EDHS) questionnaire on household asset ownership were assessed; the wealth index was computed by using principal component analysis, and the wealth status was categorized into three groups and ranked from poorest to wealthiest.

*Distance from health facility*: approximate distance of respondent's home from the nearest health centre on foot in minutes as reported by respondents were recorded and converted to hours and dichotomized as “≤2 hours” and “>2 hours”.

*Knowledge of key danger signs during childbirth*: a woman who spontaneously mentioned three or more of the four key danger signs during labor and childbirth (severe vaginal bleeding, prolonged labor (>12 hours), convulsions, and retained placenta) were categorized as having “good knowledge” and otherwise “poor knowledge.”

*Obstetric complication during delivery*: the occurrence of one or more of the following complications: prolonged/obstructed labor, excessive bleeding, mother had convulsions, breech presentation, and emergency Cesarean Section (C/S).

### 2.5. Data Management and Analysis

Data were coded and entered into EPIDATA version 3.1 to minimize logical errors and design skipping patterns and then exported to SPSS for Windows version 20 for cleaning and analysis. Descriptive statistics such as proportions, means, and standard deviation (±SD) were computed to describe the sociodemographic and economic as well as reproductive characteristics of the study participants.

To identify the determinants of perinatal mortality, first, bivariate analysis was done by cross-tabulating each explanatory variable with the outcome of interest, and all the variables having *p* < 0.25 were considered as candidates for the final multivariable binary logistic regression model. Finally, all the variables having *p* < 0.05 in the multivariable model were taken as having statistically significant associations. The adjusted odds ratios (AOR) with corresponding 95% CI were used to show the strength of the associations. During the multivariable analysis, the Hosmer and Lemeshow test of the goodness of fit was used, and it suggested the model is a good fit to the data as *p* = 0.977 (>0.05). Other assumptions, including interactions and multicollinearity at Variance Inflation Factor (VIF) <10, were also checked. However, neither interactions nor multicollinearity was detected among the considered variables.

## 3. Results

### 3.1. Magnitude of Perinatal Mortality

**Figure 1:**
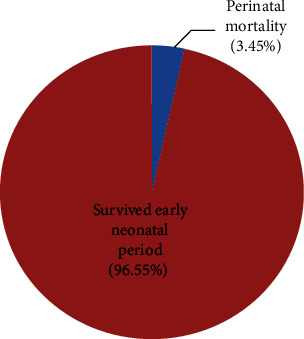
The magnitude of perinatal mortality in Jimma Zone, Southwest Ethiopia, 2017.

Among the 3474 pregnancies lasting 28 weeks or more and had been on follow-up, there were 120 perinatal deaths, making a perinatal mortality rate of 34.5 (95% CI: 28.9, 41.1) deaths per 1000 births. Of these, 47 were stillbirths, with a stillbirth rate of 13.5 (95% CI: 10.2, 17.9), and 73 were early neonatal deaths, with an early neonatal mortality rate of 21.0 (95% CI: 16.7, 26.3) (Figure 1).

### 3.2. Sociodemographic Characteristics of Respondents of the Case-Control Study

Most, 370 (77.1%), of the mothers were from rural residents, 102 (85%) among cases and 268 (74.4%) among controls. Two-thirds, 317 (66%), were in the age range of 20-29 years, 87 (72.5%) among cases and 230 (63.9%) among controls. The majority, 264 (55%), of the mothers were illiterates, 74 (61.7%) of the cases and 190 (52.8%) of the controls. Nearly nine-in-ten, 422 (87.9%), belongs to the Oromo ethnic background and Muslim by religion, 415 (86.5%). Most of the mothers, 448 (93.3%), were housewives, and most of the husbands, 339 (70.6%), were farmers by occupation (Table 1).

### 3.3. Determinants of Perinatal Mortality

In the bivariate analysis, sociodemographic characteristics, such as ethnicity, religion, occupation, age, education, income, place of residence, and distance from a health facility, were considered. But only the last five variables had *p* < 0.25 and included in the multivariate analysis. Maternal service-related variables such as ANC visit, birth preparedness and complication readiness plan, knowledge of key danger signs, and skilled care at birth were also considered, and all had *p* < 0.25 except birth preparedness and complication readiness. Variables related to the condition of pregnancy and labor such as parity, types of birth (singleton or twins), previous history of perinatal mortality, and occurrence of complications during labor were considered, and all had *p* < 0.25 and included in the final multivariate logistic regression model.

However, after adjustment in the multivariable logistic regression analysis, all the sociodemographic and economic variables had a nonsignificant association with perinatal mortality, whereas ANC visit, knowledge of key danger signs, skilled care at birth, parity, types of birth, previous history of perinatal mortality, and occurrence of complications during labor had a statistically significant association with perinatal mortality at *p* < 0.05.

Having at least 1-3 ANC visits (AOR = 0.50; 95% CI: 0.29, 0.86) as well as 4 or more visits (ANC4+) (AOR = 0.46; 95% CI: 0.23, 0.91) were significantly associated with a reduction of perinatal mortality as compared with not using ANC at all. Having good knowledge of key danger signs during pregnancy was also significantly associated with a reduction of perinatal mortality (AOR = 0.27, 95% CI: 0.10, 0.75) as compared with having poor knowledge. Births attended at a health facility by skilled attendants have lower odds of perinatal mortality as compared to those attended at home by unskilled personnel (AOR = 0.34, 95% CI: 0.19, 0.61).

Primipara and grand multipara mothers had more than three times (AOR = 3.38, 95% CI: 1.90, 6.00) and nearly two times (AOR = 1.83, 95% CI: 1.12, 3.14) higher risk of having perinatal mortality as compared with multiparous mothers having 2-4 births, respectively. Mothers having twin pregnancy had increased odds of occurrence of perinatal mortality by more than five times as compared with a singleton pregnancy (AOR = 5.29, 95% CI: 1.46, 19.21). Mothers who had a previous history of perinatal mortality were more than three times at a higher risk of experiencing perinatal mortality (AOR = 3.33, 95% CI: 1.27, 8.72). Similarly, mothers experiencing any of the obstetric complications during labor had more than four times higher risk of having perinatal mortality (AOR = 4.27; 95% CI: 2.40, 7.59) (Table 2).

## 4. Discussion

This community-based study determined the magnitude of perinatal mortality and tried to identify its determinants related to maternal health care use during pregnancy and childbirth as well as other determinants related to the conditions of pregnancy and labor.

The magnitude of perinatal mortality in this study was 34.5 deaths per 1000 births. This is comparable with the findings of the Ethiopian Demographic and Health Survey (EDHS) 2016, which were 33 deaths per 1000 births [5]. But this finding is higher than the finding of a study conducted in North Shoa, Ethiopia, 16.5 deaths per 1000 births [10]. This might be due to the setting deference, as North Shoa is relatively closer to the center of the country and has better access to health care as compared to Jimma Zone, Southwest Ethiopia, where most communities live in remote districts. In this study, early neonatal deaths accounted for 73 (60.8%) of the 120 perinatal deaths. This points to the need for special care during the immediate newborn and the first 7 days of life.

In this study, attending ANC during pregnancy was found to reduce perinatal mortality by more than half. This finding is also reported in a study done at Jimma University Specialized Hospital, Ethiopia, and a study done in Tigray, Ethiopia [6, 9]. This may be due to the fact that health problems that possibly may lead to either stillbirth or early neonatal death can be identified and treated timely during ANC visits. Similarly, the perinatal mortality in this study was about 70% lower among mothers having good knowledge of key danger signs during childbirth as compared with those with poor knowledge. This finding is also consistent with other previous studies [10, 11]. This may be due to the reason that women who have a better knowledge of key danger signs are more likely to use health care during pregnancy and childbirth. They are also more likely to seek treatment when the danger sign happens before they lead to stillbirth or early neonatal death.

Using skilled care at birth was also found to reduce perinatal mortality by about two-thirds. This finding is also in line with the findings of studies conducted in Ethiopia and other countries [6, 9, 12–15]. This can be explained by the fact that when births are attended by skilled personnel, the complications leading to stillbirth or early neonatal death, including obstructed labor, can be detected and managed or referred in a timely manner.

In this study, primipara mothers and grand multipara mothers were about three times and two times more likely to have perinatal mortality as compared to multiparous mothers, having 2-4 births, respectively. This has been reported in a study conducted in India and West Gojjam Zone, Ethiopia [15, 16]. This may be due to the reason that primipara mothers and grand multipara mothers are more likely to experience complications during pregnancy as well at birth, including obstructed and prolonged labor leading to either stillbirth or early neonatal death. This will have a programmatic implication of giving special emphasis and care to primipara mothers and grand multipara mothers during ANC and labor monitoring.

Twin pregnancies were more than five times at risk of ending in perinatal mortality. This is also consistent with the findings of studies conducted in North Shoa, Ethiopia [9], in India [15], and in Portugal [17]. This can be explained by the fact that mothers with a twin pregnancy are more likely to face complications during pregnancy as well as childbirth, including obstructed labor and birth asphyxia which leads to either stillbirth or early neonatal death. This also has a programmatic implication of giving special care for women with a twin pregnancy during ANC as well as intrapartum care.

In this study, mothers with a previous history of perinatal mortality were more than three times more likely at risk of experiencing perinatal mortality. Previous studies also revealed similar findings [12, 16]. This might be due to the reason that women who have repeated stillbirth or early neonatal mortality may have biological or anatomical problems related to the uterus, pelvic, or any birth canal that may lead to complications and lead to perinatal mortality. This also indicates the programmatic implication of giving special investigations and care for women having a history of perinatal mortality during pregnancy and labor to avert subsequent occurrence.

Women facing any one of the obstetric complications during labor were more than four times more likely to have perinatal mortality. This finding is also consistent with the findings of previous studies [6, 9–14, 18]. This may indicate that maternal complications like bleeding, obstructed/prolonged labor, and others can also have an effect on the survival of the fetus. This also points to the special and strict follow-up of fetal conditions of mothers with any obstetric complications.

This study may have its own limitations of recall bias because of the case-control nature of the study design. Sociodemographic characteristics such as wealth index and educational status had a statistically nonsignificant association in this study while they had a significant association in other studies. This may be due to the low number of a high level of education and a high level of income as most are rural residents. Hence, all readers have to take these limitations into consideration while using the finding of this article.

## 5. Conclusions

The magnitude of perinatal mortality is high as compared to the national target of attaining a stillbirth rate of <10 per 1000 births and neonatal mortality rate of <10 per 1000 live births by 2020. Care during pregnancy, knowledge of key danger signs, skilled care at birth, parity, twin pregnancy, previous history of perinatal mortality, and occurrence of complications during labor were identified as determinants of perinatal mortality. Thus, interventions need to focus on increasing knowledge of key danger signs and improving the utilization of skilled care during pregnancy and delivery. Special emphasis and care also need to be given to mothers with a previous history of perinatal mortality, twin pregnancies, and encountering any form of obstetric complication during labor.

## Figures and Tables

**Table 1:** Sociodemographic characteristics of the respondents for the case-control study, Jimma Zone, Southwest Ethiopia.

Variable	Cases (*n* = 120) *n*(%)	Controls (*n* = 360) *n*(%)	Total (*n* = 480) *n*(%)
Place of residence			
Urban	18 (15.0)	92 (25.6)	110 (22.9)
Rural	102 (85.0)	268 (74.4)	370 (77.1)
Age of mothers (years)			
17-19	4 (3.3)	15 (4.2)	19 (4.0)
20-29	87 (72.5)	230 (63.9)	317 (66.0)
30-42	29 (24.2)	115 (31.9)	144 (30.0)
Educational status of mothers			
Illiterate (no formal education)	74 (61.7)	190 (52.8)	264 (55.0)
Primary school (grades 1-8)	39 (32.5)	139 (38.6)	178 (37.1)
Secondary school (grades 9-12)	6 (5.0)	23 (6.4)	29 (6.0)
Tertiary (college/university)	1 (0.8)	8 (2.2)	9 (1.9)
Ethnicity of mothers			
Oromo	110 (91.7)	312 (86.7)	422 (87.9)
Dawuro	5 (4.2)	15 (4.2)	20 (4.2)
Amhara	1 (0.8)	16 (4.4)	17 (3.5)
Gurage	2 (1.7)	9 (2.5)	11 (2.3)
Others^∗^	2 (1.7)	8 (2.2)	10 (2.1)
Religion of mothers			
Muslim	109 (90.8)	306 (85.0)	415 (86.5)
Orthodox Christian	6 (5.0)	43 (11.9)	49 (10.2)
Protestant	5 (4.2)	11 (3.1)	16 (3.3)
Mothers' occupation			
Housewife	111 (92.5)	337 (93.6)	448 (93.3)
Government employed	4 (3.3)	9 (2.5)	13 (2.7)
Others^++^	5 (4.2)	14 (3.9)	19 (4.0)
Husbands' occupation			
Farmer	95 (79.2)	244 (67.8)	339 (70.6)
Government employed	8 (6.7)	46 (12.8)	54 (11.3)
Merchant	10 (8.3)	43 (11.9)	53 (11.0)
Daily laborer	6 (5.0)	23 (6.4)	29 (6.1)
Student	1 (0.8)	4 (1.1)	5 (1.0)

^∗^Kafa, Tigre, Yem; ^++^student, daily laborer, merchant.

**Table 2:** Determinants of perinatal mortality in Jimma Zone, Southwest Ethiopia.

Variables	Perinatal mortality		Crude OR (95% CI)	Adjusted OR (95% CI)
	Cases (*n* = 120) *n*(%)	Controls (*n* = 360) *n*(%)		
Place of residence				
Rural	102 (85.0)	268 (74.4)	1.00	1.00
Urban	18 (15.0)	92 (25.6)	0.51 (0.30, 0.90)	0.92 (0.39, 2.18)
Age of mother				
< 20 years	4 (3.3)	15 (4.2)	1.00	1.00
≥ 20 years	116 (96.7)	345 (95.8)	1.27 (0.41, 3.88)	1.75 (0.49, 6.21)
Maternal education				
No formal education	74 (61.7)	190 (52.8)	1.00	1.00
Primary (grades 1-8)	39 (32.5)	139 (38.6)	0.72 (0.46, 1.12)	0.92 (0.54, 6.21)
Secondary or above (≥9th)	7 (5.8)	31 (8.6)	0.58 (0.25, 1.37)	1.23 (0.40, 3.81)
Wealth index (tertiles)				
Poorest	55 (45.8)	143 (39.7)	1.00	1.00
Middle	28 (23.4)	66 (18.4)	1.10 (0.64, 1.90)	1.28 (0.68, 2.41)
Richest	37 (30.8)	151 (41.9)	0.64 (0.40, 1.03)	0.84 (0.48, 1.47)
Distance from health facility				
≤ 2 hours	10 (8.3)	59 (16.4)	1.00	1.00
> 2 hours	110 (91.7)	301 (83.6)	2.16 (1.07, 4.36)	1.43 (0.50, 4.14)
ANC visit				
No ANC at all	51 (42.5)	88 (24.4)	1.00	1.00
1-3 visits	45 (37.5)	154 (42.8)	0.50 (0.31, 0.81)	0.50 (0.29, 0.86)
4 or more visits	24 (20.0)	118 (32.8)	0.35 (0.20, 0.61)	0.46 (0.23, 0.91)
Knowledge of danger signs				
Poor knowledge	115 (95.8)	294 (81.7)	1.00	1.00
Good knowledge	5 (4.2)	66 (18.3)	0.67 (0.43, 1.06)	0.27 (0.10, 0.75)
Birth attendant				
Nonskilled attendant	90 (75.0)	204 (56.7)	1.00	1.00
Skilled birth attendant	30 (25.0)	156 (43.3)	0.44 (0.27, 0.69)	0.34 (0.19, 0.61)
Parity				
Primipara (1st birth)	35 (29.2)	61 (16.9)	2.61 (1.56, 4.37)	3.38 (1.90, 6.00)
2-4 births	51 (42.5)	232 (64.4)	1.00	1.00
> 4 births	34 (28.3)	67 (18.6)	2.31 (1.38, 3.85)	1.83 (1.12, 3.14)
Type of birth				
Singleton	109 (90.8)	355 (98.6)	1.00	1.00
Twins	11 (9.2)	5 (1.4)	7.17 (2.44, 21.07)	5.29 (1.46, 19.21)
Previous history of perinatal mortality				
No	107 (89.2)	350 (97.2)	1.00	1.00
Yes	13 (10.3)	10 (2.8)	4.25 (1.81, 9.97)	3.33 (1.27, 8.72)
Complication during labor				
No	77 (64.2)	304 (84.4)	1.00	1.00
Yes	43 (35.8)	56 (15.6)	3.03 (1.90, 4.85)	4.27 (2.40, 7.59)

## Data Availability

All related data used to support the findings of this study are included within the manuscript, and the SPSS data set is available from the author and can be obtained on request at gurmesatura@gmail.com.
